# Kid-size robot humanoid walking with heel-contact and toe-off motion

**DOI:** 10.7717/peerj-cs.797

**Published:** 2022-03-15

**Authors:** Yucong Wu, Yang Pan, Xiaokun Leng, Zhicheng He

**Affiliations:** 1 Shenzhen Key Laboratory of Biomimetic Robotics and Intelligent Systems, Department of Mechanical and Energy Engineering, Southern University of Science and Technology, Shenzhen, China; 2 Guangdong Provincial Key Laboratory of Human-Augmentation and Rehabilitation Robotics in Universities, Southern University of Science and Technology, Shenzhen, China

**Keywords:** Humanoid robot, LIPM, Biped robot, Heel-contact and Toe-off

## Abstract

Human-like features, like toe-off, heel-strike can enhance the performance of bipedal robots. However, few studies have considered the anthropomorphism of walking planning. Fewer studies have achieved their toe-off, heel-strike gait planning framework in a child-sized humanoid robot platform. This paper presents a human-like walking control framework based on the Divergent Component of Motion (DCM) com planning method that enables a child-sized humanoid robot to walk with a humanoid pattern with a speed of 0.6 s per step a strike of 30 cm. The control framework consists of three parts: the human-like gait generation of the center of mass (CoM) and swings foot trajectory, the dynamic replan in phase switch and the upper body stabilization controller. The dynamic replanning of the CoM and foot trajectory can efficiently decrease the vibration in the step-phase switch. The up-body stabilization controller can reduce the up-body swing in walking and increase the robot's stability while walking. The robot uses a mems-based inertial measurement unit (IMU) and joint position encoders to estimate the current state of the robot and use force-sensitive resistors (FSR) on the robot foot to identify the actual step phase of the robot. None of these solutions is high-cost or difficult to integrate with a child-size robot. Software simulations and walking experiments are using to verify the motion control algorithm. The effectiveness of the pattern generation and the controller can realize more human-like walking styles in a child-size robot are confirmed.

## Introduction

As we know, the walking pattern generation and control of biped robots have been an ongoing research hotspot in recent years. Many researchers use the linear inverted pendulum (LIP) as the model for the gait generation algorithm to utilize the stable walking trajectory. Nevertheless, the Hypothesis of LIP requires the center of mass (CoM) of the robot to stay at the same height, which is why the robot must walk with a bended knee. On the other hand, many people expressed the criticism that this kind of walking is not human-like.

Many researchers have tried to solve this problem by demonstrating using their separate methods. Ogura et al. (2006) designed a humanoid robot, WaBIAN-2R, with two passive joints in its toe to utilize a more human-like walking pattern. Lohmeier (2010) is another humanoid robot with 4 joints in each foot designed by Lohmeier (2010) and some other researchers like Li et al. (2010) and Kurazume et al. (2005) and other researchers (Kim et al., 2008; Morisawa et al., 2005; Sekiguchi et al., 2006) have also realized good looking human-like biped locomotion by real robots. However, their works rely on special mechanisms of the robot’s feet. Nevertheless, these kinds of designs are not easy to implement in a child-size humanoid robot. In recent years, some scholars (Carpentier et al., 2016; Feng et al., 2013; Kuindersma et al., 2016; Leng et al., 2020; Wieber, 2006) are also adopting optimization-based methods to achieve robot walking. However, the versatility of these methods is outstanding, but the implemented gait algorithm is not universal and may consume more time when calculating.

**Figure 1:**
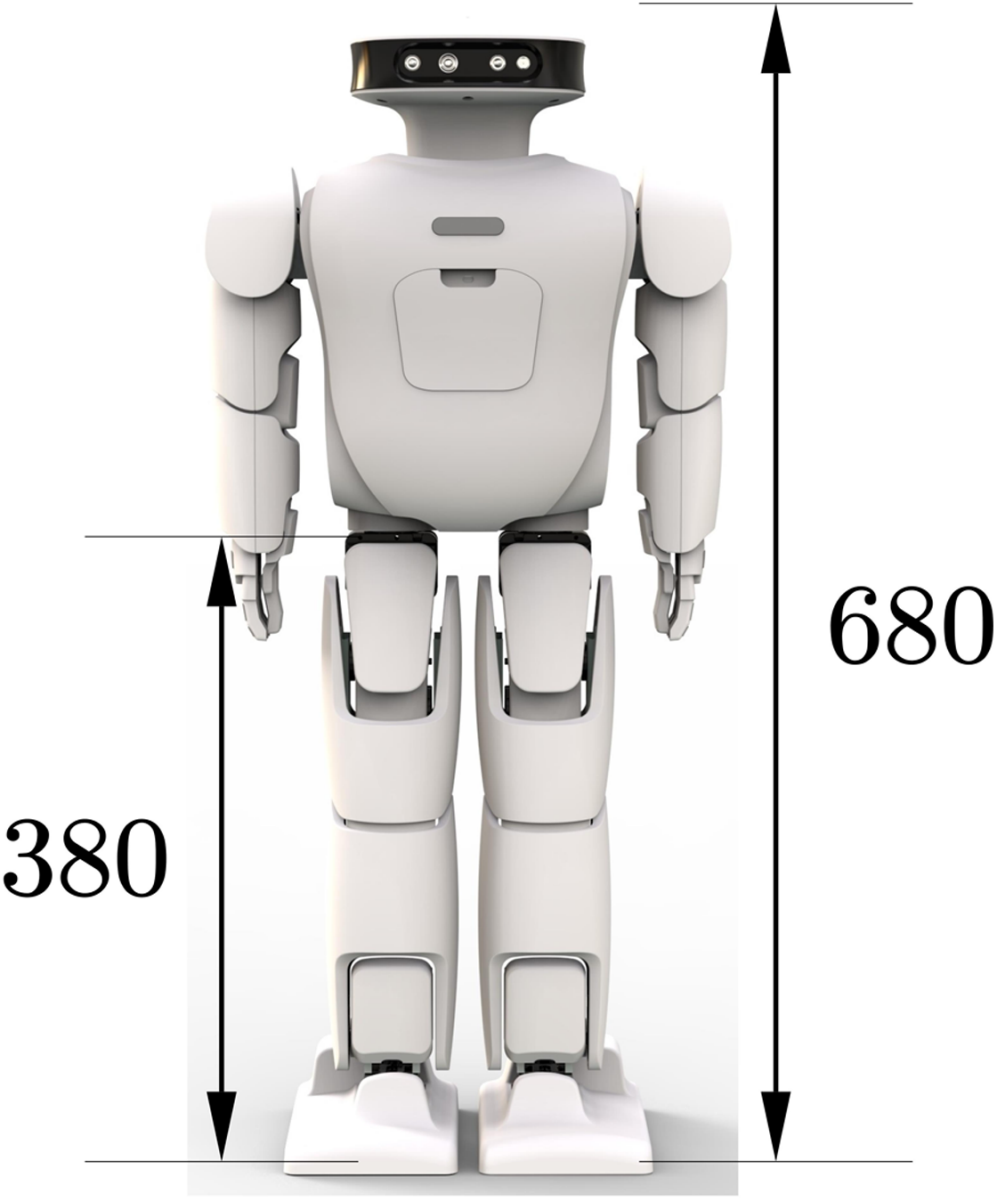
Roban humanoid robot.

This paper presents a human-like walking control framework based on the DCM theory. This framework includes a footprint generation unit that can generate footprints according to the robot’s motion instructions. The robot can dynamically generate the CoM and foot trajectory from the given footprint position. Considering the disturbance during walking, a robot stability controller is implemented to improve the robot’s stability during operation. We validate our approach in physically realistic simulations and use the Roban child-sized humanoid robot with a height of about 68 cm. From Fig. 1, we can see the specific dimensions of the experimental robot used.

This paper is organized as follows. The “Methods” section describes the basic theory of the gait trajectory generation of a biped robot based on the capture point (CP) theory. On this basis, we introduce the modified CoM trajectory and foot trajectory generation methods needed to realize the human-like gait in the prospect. The “Stablizer” section presents an overall robot human-like walking control framework, followed by an event-based switching mode of one-foot and two-foot support. Finally, a stable controller for a child-size robot with a low-cost modular actuator is proposed. The “Experiments” section constructed the overall robot dynamics simulation model and verified the corresponding human-like gait control algorithm in the simulation and the real object.

## Methods

### Pattern generation

#### Linear inverted pendulum and capture point

The linear inverted pendulum (LIP) model is a major dynamic model used for domestic modeling of biped robot walking (Choi et al., 2007; Shuuji et al., 2010; Sakagami et al., 2002; Sugihara, 2009). The following assumptions must be met:

1. The robot is seen as a mass point and a massless light rod
2. CoM of the robot is held at constant height *Z*

**Figure 2:**
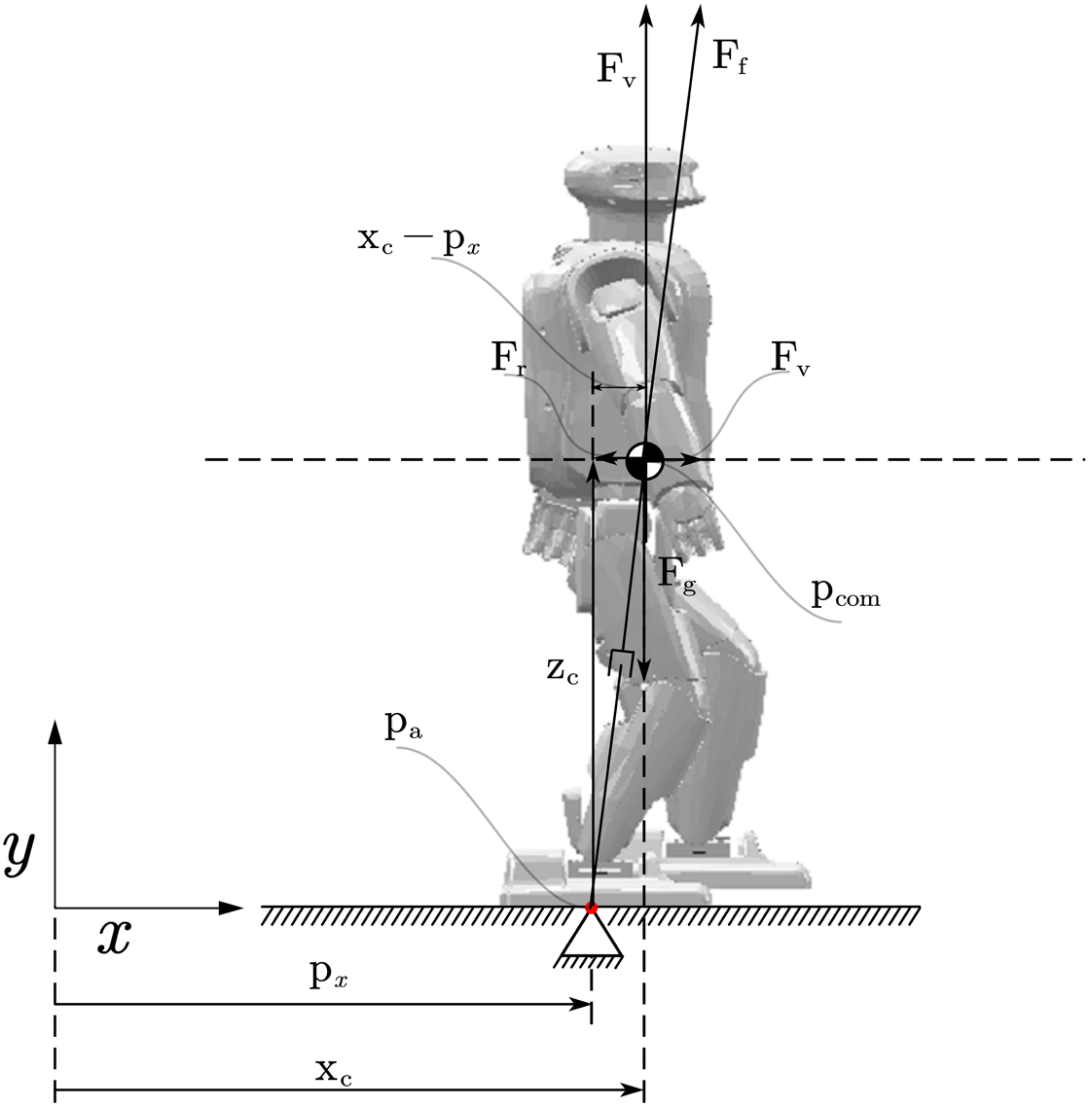
Linear inverted pendulum model. Table for notations (Notation & Description). Z_c_: The robot’s Center of Mass in *x* axis; F_f_: The ground reaction force; F_v_: The vertical component of F_f_; P_com_: The robot’s Center of Mass position; P_a_: Total moment acting on the CoM; X_c_: The robot’s Center of Mass in *x* axis; p_z_: The robot’s Zero Moment Point.

Under the premise of the above linear inverted pendulum model, the motion mode of the center of mass of the robot is decoupled in the front and rear motion direction and the left and right motion direction. Therefore, the motion patterns in the center of mass of the robot in these two directions during walking can be considered separately. Figure 2 gives an overview of the whole dynamic model. The ground reaction force *F*_*f*_ is collinear with the vector $\left( {{P_{{\rm com}}} - {P_{\rm a}}} \right)$. *F*_*v*_ is the vertical component of *F*_*f*_. It compensates for the gravitational force *F*_*g*_ acting on the CoM. By comparison of the force parallelogram and the geometrical parallelogram we find

(1) $$\displaystyle{{{F_h}} \over {{F_v}}} = \displaystyle{{{F_r}} \over {{F_g}}} = \displaystyle{{m{{\ddot x}_c}} \over {mg}} = \displaystyle{{{x_c} - {p_x}} \over {{z_c}}}$$

Therefore, an expression for the horizontal acceleration of the CoM is

(2) $${\ddot x_c} = {\omega ^2}\left( {{x_c} - {p_x}} \right)$$

where $\omega = \sqrt {g/{z_c}}$ and px is the x-coordinate of the Zero-Moment-Point (ZMP). *ω* has to be positive in this paper. The complete system dynamics of the LIP model is the following equation

(3) $$\dot \sigma = \left[ {\matrix{ 0 & 1 \cr {{\omega ^2}} & 0 \cr } } \right]\sigma + \left[ {\matrix{ 0 \cr { - {\omega ^2}} \cr } } \right]{p_x}$$

where $\sigma = {\left[ {{x_c},{{\dot x}_c}} \right]^T}$. The analytical solution of (3) is

(4) $$\sigma (t) = \left[ {\matrix{ {\cosh (\omega t)} & {\displaystyle{1 \over \omega }\sinh (\omega t)} \cr {\omega \sinh (\omega t)} & {\cosh (\omega t)} \cr } } \right]{\sigma _o} + \left[ {\matrix{ {1 - \cosh (\omega t)} \cr { - \omega \sinh (\omega t)} \cr } } \right]{p_x}$$

Pratt et al. (2006) and Hof, Gazendam & Sinke (2005) independently introduced the Capture Point (CP). The Capture Point is a point on the ground where the robot has to step to complete rest, which means that the center of mass (CoM) can fully stop horizontally at that point. For a general robot state $\sigma = {\left[ {{x_c},{{\dot x}_c}} \right]^T}$ it is defined as

(5) $${\xi _x} = {x_c} + \displaystyle{{{{\dot x}_c}} \over \omega }$$

Since we have the definition of Capture point. We need to derive the dynamics based on the capture point theory. Solving (5) for ${\dot x_c}$ we can get

(6) $${\dot x_c} = - \omega \left( {{x_c} - {\xi _x}} \right)$$

We find that ${\dot x_c}$ has a stable first-order open loop dynamics with time constant $\displaystyle{1 \over \omega }$. By differentiation (5) and (6) we know that

(7) $${\dot \xi _x} = {\dot x_c} + \displaystyle{{{{\dot x}_c}} \over \omega } = \omega \left( {{\xi _x} - {p_x}} \right)$$

**Figure 3:**
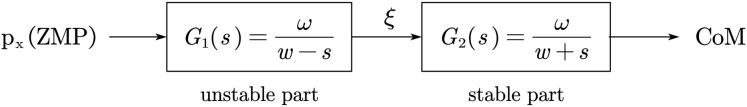
Capture point model.

The Capture Point *ξ*_*x*_ has an unstable first-order open loop dynamics. Figure 3 show the coupling of the two states ${{\rm {\cal X}}_C}$ and *ξ*_*x*_. By considering (6) and (7) we find the systems dynamics is

(8) $$\dot \theta = \left[ {\matrix{ { - \omega } & \omega \cr 0 & \omega \cr } } \right]\theta + \left[ {\matrix{ 0 \cr { - \omega } \cr } } \right]{p_x}$$

where $\omega = \sqrt {g/{z_c}}$ , $\theta = {\left[ {{x_c},{\xi _x}} \right]^T}$ and *p*_*x*_ is the ZMP.

#### Step planner

In the process of planning the robot’s walking by the upper-level robot motion controller, it is usually only the target position and posture of the robot. Therefore, a footprint generator is needed to convert the target pose difference data into the target footprint data of the biped robot. For example, the forward movement of the syncline needs to be gradually given the footprint data of the forward movement of the syncline, and the CP trajectory planner introduced below requires the information of the last few footprints to plan a better centroid trajectory. We expect a walking framework that can continuously plan the robot’s trajectory rather than intelligently let the robot walk a certain number of steps. For biped robots, the usual planning goal of the upper planner is to run from the current position to another given target position, so the corresponding motion relationship is often given in the form of required increments. The motion parameters given by the upper-level planning can be expressed as (*dx*, *dy*, *θ*). Several planning examples are showing below:

(1) Forward/backward step planning

**Figure 4:**
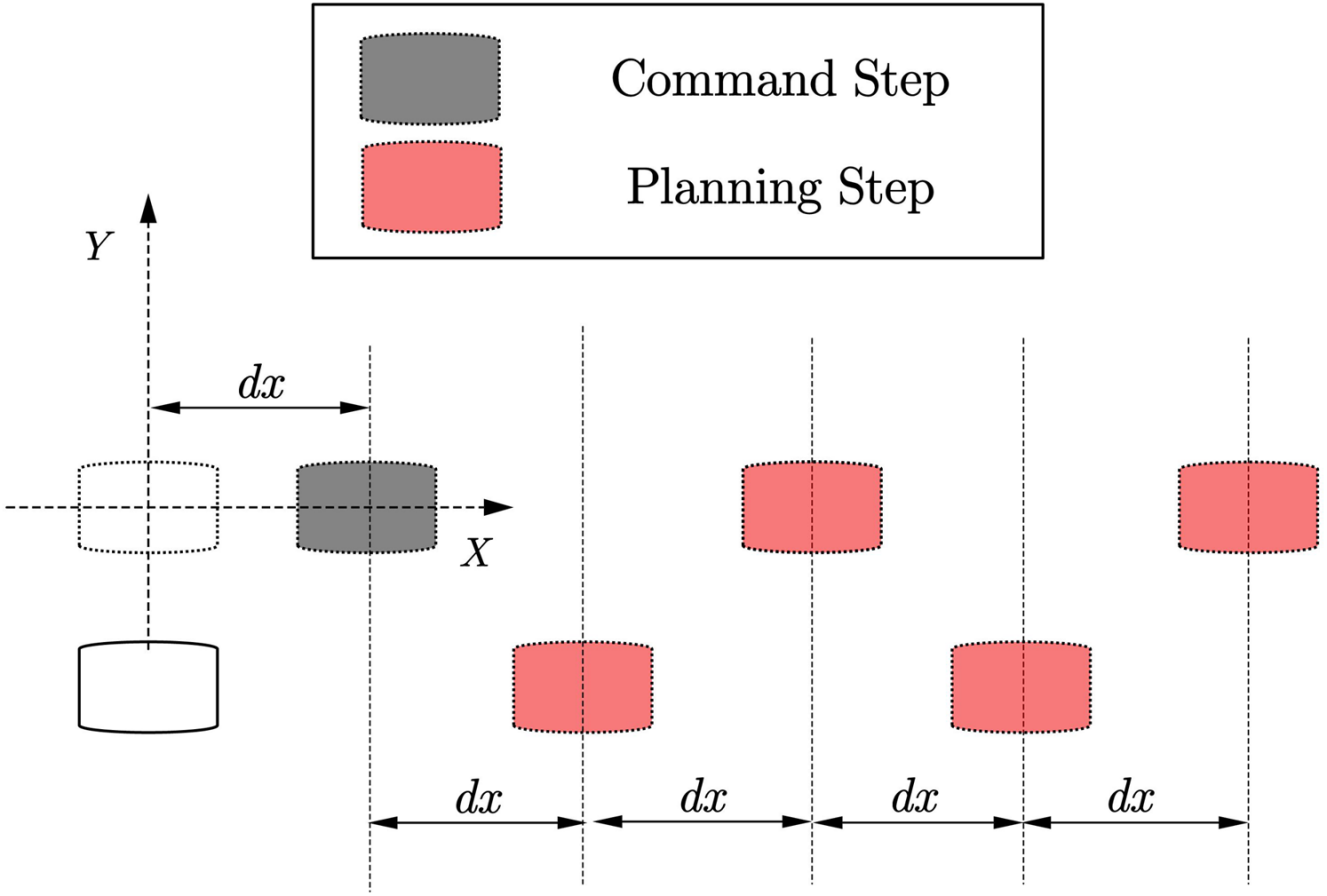
Forward step planning.

As shown in Fig. 4. The forward/backward trajectory planning algorithm is relatively simple. The black steps are the footprints calculated directly through the given running instructions, and the red footprints are assumed to be a series of steps generated by the robot according to the gait pattern of the robot walking footprints. According to the subsequent centroid trajectory generator, it can be found that when the number of footprints in the supplementary plan reaches (4), the first few single foot support phase trajectories in the overall centroid trajectory generated by these footprints are basically the same. Special, For the forward or backward gait, the trajectory of the center of mass in the one-foot support phase is symmetric about the *xoz* plane.

(2) Side step planning

**Figure 5:**
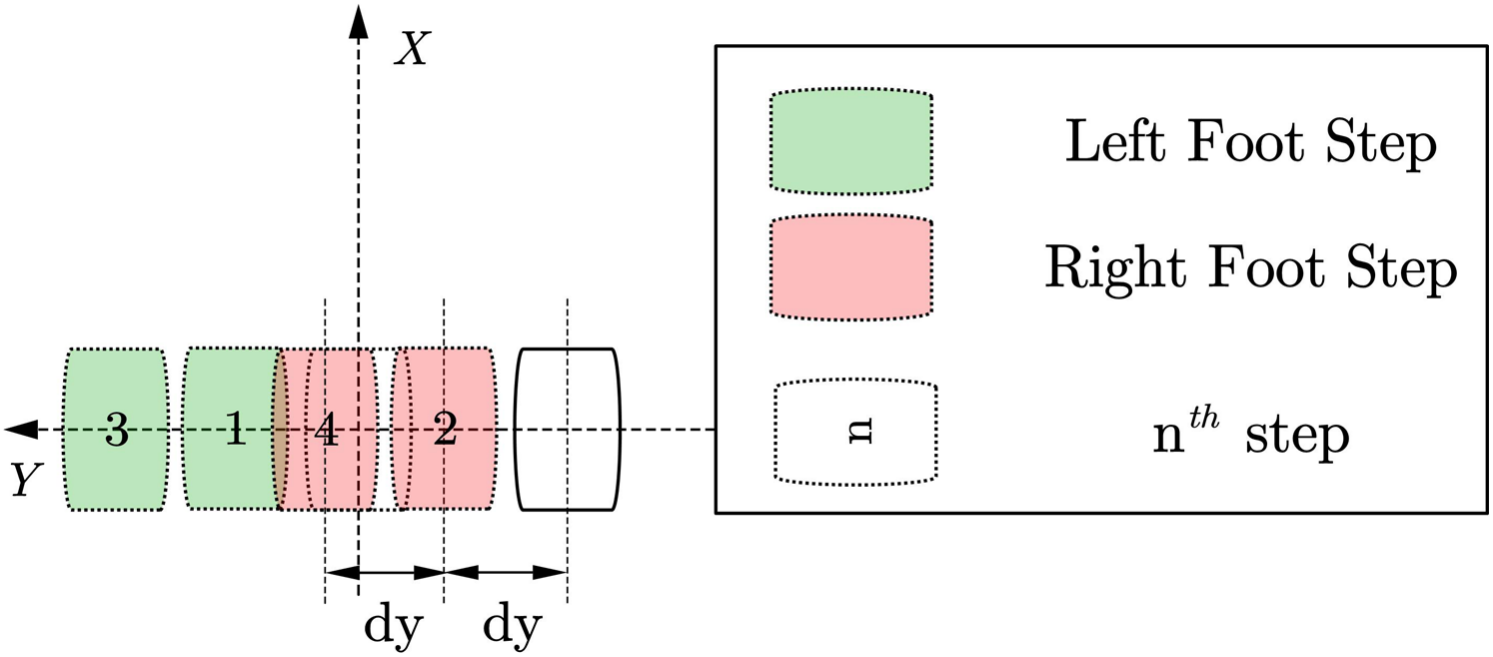
Side walk planning.

As shown in Fig. 5. For the robot footprint planning required for the side shift of the biped robot, in addition to the footprints that need to generate symmetrical footprints, it is also necessary to consider the interference problem of the swinging feet that may be caused in the subsequent trajectory planning process. A simplified strategy is for the lateral direction. To run the command, we need to move the foot in which direction first and then move the foot in the other direction. At the same time, the other foot is resting on the foot according to the planned foot gap.

(3) Steering step planning

**Figure 6:**
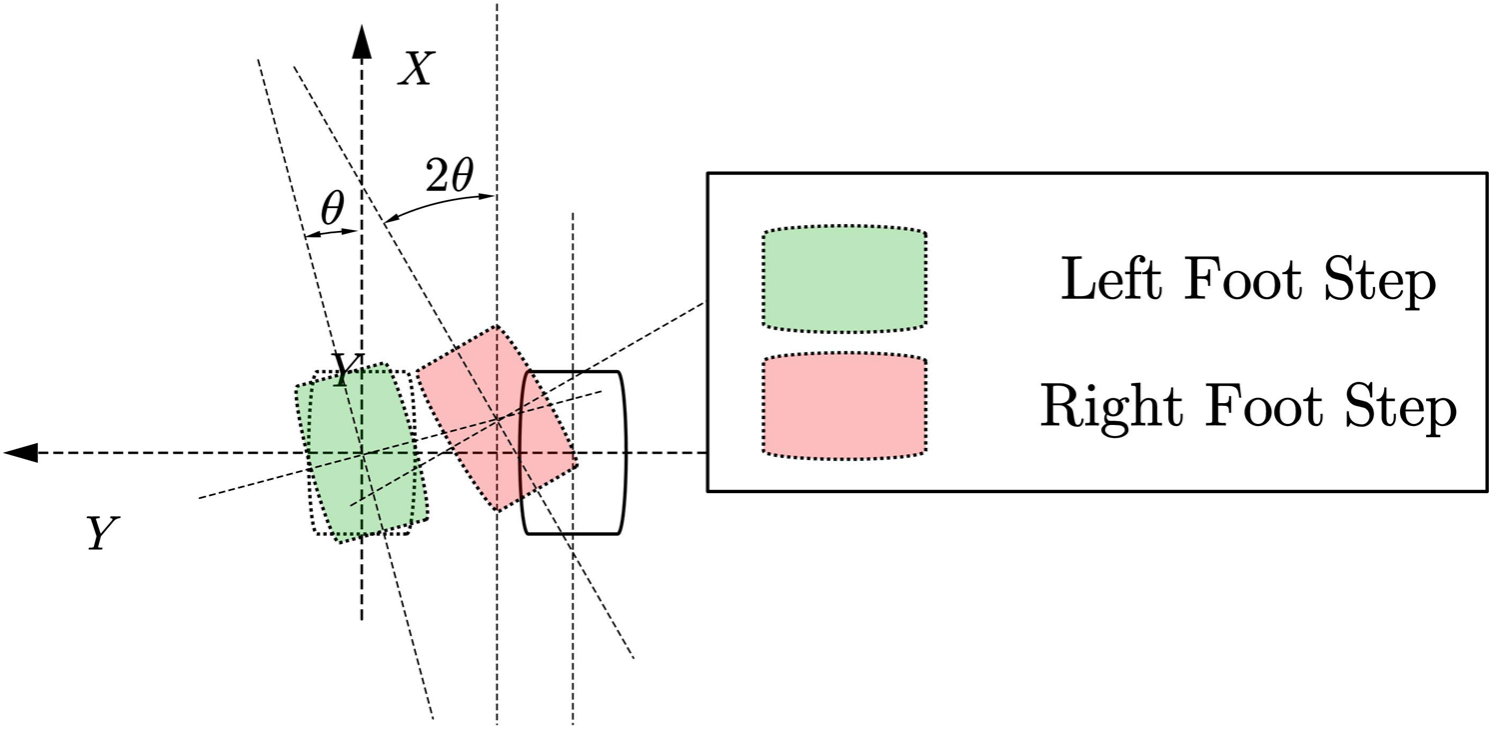
Steering step planning.

As shown in Fig. 6. The footprint generation of the steering gait algorithm is similar to the generation of the side-shift gait, and it is also necessary to consider the problem of footprint interference to determine which foot to start from. In addition, to achieve more block steering, the robot’s feet will turn during the operation. This planning method will more easily cause interference between the two feet. This situation will be checked in the gait planner to avoid interference during the actual operation of the robot. If the footprints are generated, When the detector detects that the footprint will interfere, it will limit the steering angle to a range that does not cause interference.

(4) Mix step planning

**Figure 7:**
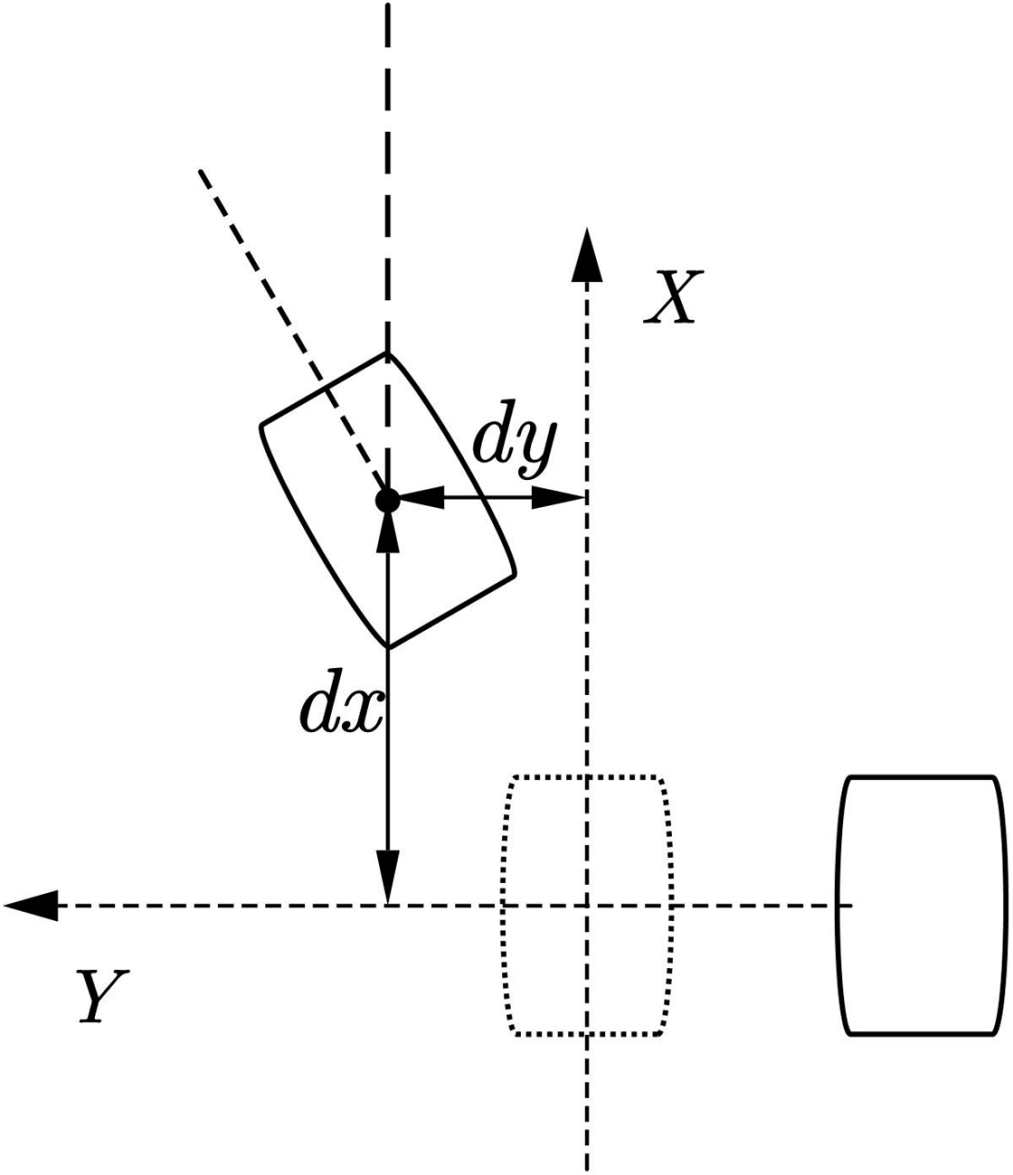
Arbitrary step planning.

As shown in Fig. 7, combining the above-fixed gait pattern generation, the footprint generator we proposed can essentially generate corresponding footprints for any form of command position incremental input. For example:

1. It can use both forward walking and side walking at the same time. Features, plan a trajectory that allows the robot to walk obliquely forward.
2. The characteristics of simultaneous side walking and turning can be used to allow the robot to achieve a larger angle of turn.

#### CoM trajectory generator

In common biped robot application scenarios, such as Englsberger et al. (2011), the usual method is first to generate a reference trajectory of the CP point and then implement a feedback controller so that the CP point of the actual robot can track the reference trajectory of the CP point. However, this study did not use the methods in the references. Since the experimental platform used in this article is a child-size humanoid robot. Suppose we directly use the method described in the literature to implement it. In that case, the following two problems will arise: On the one hand, the experimental robot we use is a module. The resolution and accuracy of the encoder of the modified drive joints are not very high, resulting in more incredible noise when reading the joint angles. On the other hand, we are using a MEMS-based IMU, and the obtained linear acceleration and angular velocity noise are relatively large. Due to the above two problems, the robot will have much noise in its speed estimation, so there will be much noise in the estimation of the cp point. In this research, we further generate the desired CoM trajectory through the CP point trajectory and then calculate the joint angle of the robot through the inverse kinematics solution of the robot.

The CP point correlation theory is an inference about the linear inverted pendulum correlation algorithm. The definition of CP point is described above. The CP point correlation theory can be used to calculate the stability of the biped robot during its operation. Also, similar to the linear inverted pendulum model, we can use the theory of CP point to get the centroid trajectory of the robot operation more conveniently when the footprint position changes. Generally speaking, it is necessary to obtain the footprint information of all the steps of the robot. However, this planning method is not suitable for the situation where the robot receives continuous walking footprints.

Nevertheless, after a certain amount of footprint information (*i.e*., ZMP) sequence information is given, the centroid trajectory of the first few steps is approximately the same. Therefore, the method used is to first generate a part of the CoM trajectory sequence through the footprint generator but only use it to generate the relevant information of the first single foot support phase trajectory and discard other data in the trajectory. After actual simulation, considering the error of the trajectory and the computing power of the corresponding controller, we found that in addition to the corresponding position of the previous footprint, it is also necessary to generate the last four footprints for CoM trajectory planning.

From CP theory, we know that the relationship between the CoM position, Capture Point, and ZMP point can be expressed as

(9) $$\left[ {\matrix{ \xi \cr x \cr } } \right] = \left[ {\matrix{ {\dot \xi } \cr {\dot x} \cr } } \right] = \left[ {\matrix{ { - \omega } & \omega \cr 0 & \omega \cr } } \right]\left[ {\matrix{ \xi \cr x \cr } } \right] + \left[ {\matrix{ 0 \cr { - \omega } \cr } } \right]p$$

As we can see, (9) is a differential equation that is easy to solve. From (9), we can get

(10) $$\matrix{ {{G_2}(s):{x_{k + 1}} = {e^{ - \omega T}}\left( {{x_k} - {\xi _k}} \right) + {\xi _k}} \hfill \cr {{G_1}(s):{\xi _k} = {e^{ - \omega T}}\left( {{\xi _{k + 1}} - {p_k}} \right) + {p_k}} \hfill \cr }$$

**Figure 8:**
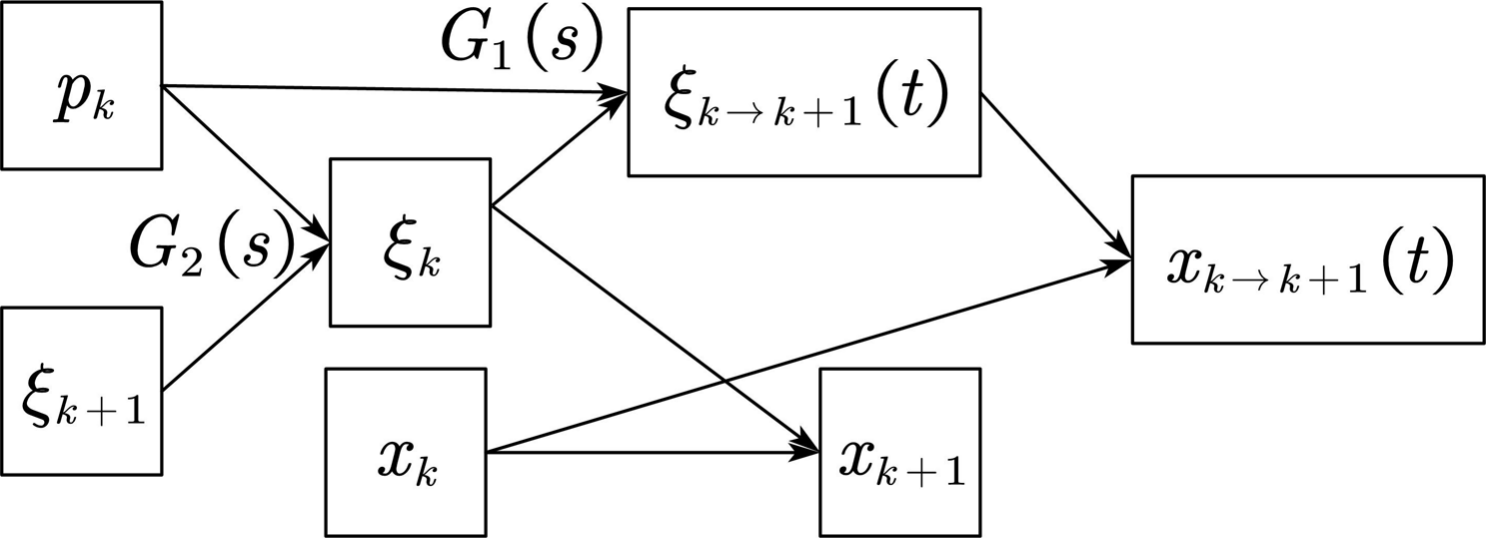
CoM recursive path.

**Figure 9:**
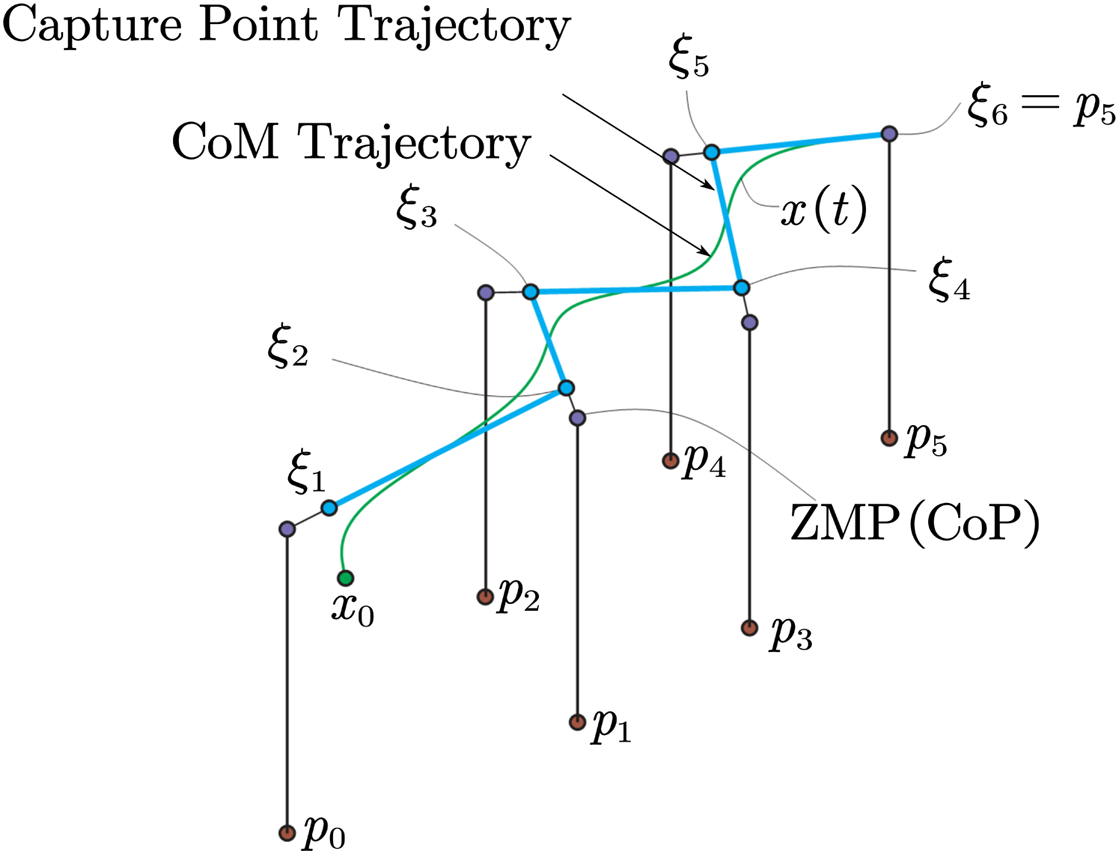
CP and CoM trajectory from ZMP.

Now we can get the expression of the CP point and the center of mass trajectory of the robot in the time domain. Therefore, as long as the initial value can be selected appropriately, the trajectory of the center of mass of the robot can be obtained when the robot is running. Since the speed of the center of mass of the robot is 0 when the robot finally stops walking, the CP point and the position of the center of mass are also coincident when it reaches that point, so it can be used as the corresponding recursive boundary condition. Through the introduction in the previous article, we can obtain four additional ZMP points that the robot is expected to pass. Therefore, we can obtain a corresponding recurrence relationship as follows:

From Fig. 8, we can see that after the boundary conditions of the last step, the previous CP point position and the position of the corresponding centroid can be cross-derived, as shown in Fig. 8 so that the entire required trajectory information can be obtained. From Fig. 9, we can know the relationship between CP trajectory, CoM trajectory and ZMP point in geometric space.

#### Variation of CoM height

**Figure 10:**
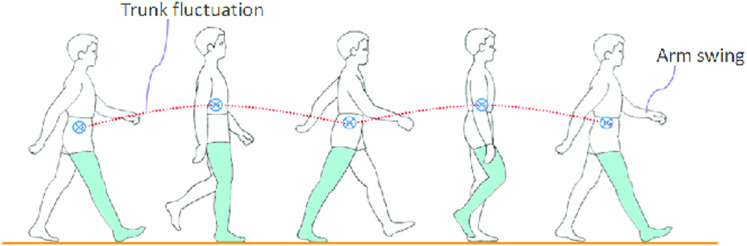
Pattern of human motion and posture in a typical walking cycle.

As we can see in the Fig. 10 (Xie et al., 2016). By studying human motion data (Chen et al., 2017; Miura et al., 2011), we found that the height of the center of mass of the human body changes during walking. Therefore, for a robot to realize a human-like gait, the CoM also needs to change z-direction. The change in the height of the robot’s center of mass can also bring some practical uses. For example, it can reduce the robot’s power consumption to a certain extent, and it can also increase the step strike when the robot is walking. However, for the trajectory planning of CoM height, the previous theory requires it to remain unchanged in the height direction. Nevertheless, we found that we can change the center of mass of the robot through a certain pattern, and when the height of the center of mass of the robot changes, we can regard the height of its center of mass as a kind of linearity. The disturbance of the inverted pendulum model, in the following, we will first analyze its impact on ZMP.

According to the derivation in the reference (Li et al., 2010), assuming that the height of the center of mass of the robot changes during walking, the corresponding ZMP point error and the center of mass motion trajectory of the corresponding robot will satisfy the following relationship:

(11) $${e_x} = {x_{ZMP}} - x_{ZMP}^{\prime} = \ddot x\displaystyle{{{z_c}\ddot z + g\left( {{z_c} - z} \right)} \over {g(\ddot z + g)}}$$

where *x* and *z* denote the 2D CoM’s position, and *g* be the gravitational constant

The value of $\ddot z$ and height *z* will affect the size of the ZMP error. Consider an acceleration variation $\ddot z \in ( - 0.2,0.2)m/{s^2}$, a height variation *z* ∈ (0.39, 0.41)*m* and an average horizontal acceleration $\ddot x = 0.8\ {\rm m}/{{\rm s}^2}$. From simulation, we can know the ZMP error is relatively small. We can also analyze its impact on the trajectory of the center of mass from another angle. It should be that we need to consider the acceleration in the Z direction when setting the CP point parameters. Therefore, the following formulas can be used to calculate the acceleration in the Z direction and without the Z direction Centroid trajectory generated during acceleration. Due to the decoupling characteristics in the X and Y directions, we use numerical calculations to analyze the influence of the centroid trajectory in the Y direction by the acceleration of the centroid in the Z direction, and its influence on the trajectory of the centroid in the Y direction is less than 5%. It is considered that the influence of acceleration in the Z direction on the trajectory at this time is significantly smaller. The value of $\ddot z$ and height *z* will affect the size of the ZMP error. Consider an acceleration variation $\ddot z \in ( - 0.2,0.2)m/{s^2}$, a height variation *z* ∈(0.39, 0.41)*m* and an average horizontal acceleration $\ddot x = 0.8\ {\rm m}/{{\rm s}^2}$. From the simulation, we can know that the ZMP error is relatively small. We can also analyze its impact on the trajectory of the center of mass from another angle. We need to consider the acceleration in the Z direction when setting the CP point parameters. Therefore, the following formulas can be used to calculate the acceleration in the Z direction and without the Z direction Centroid trajectory generated during acceleration. Due to the decoupling characteristics in the X and Y directions, we use numerical calculations to analyze the influence of the centroid trajectory in the Y direction by the acceleration of the centroid in the Z direction and its influence on the trajectory of the centroid in the Y direction is less than 5%. It is considered that the influence of acceleration in the Z direction on the trajectory at this time is significantly smaller.

It can be seen from the analysis of the above two angles. From the perspective of ZMP error, for the small acceleration of the center of mass of the robot in the Z direction, the change in ZMP is small, and the foot length of the robot is 160 mm, which can tolerate the ZMP error of the robot. From the point of view of CP generating CoM trajectory, it has little effect on the generated horizontal centroid trajectory. Therefore, it is stable for robot walking if we can design a trajectory that does not have too much displacement and acceleration in the Z direction. The impact of stability is also limited.

From the analysis in the previous article, it can be seen that the primary influence on the new walking stability of the robot is the acceleration of the robot’s center of mass in the Z direction. Therefore, we need to design a trajectory with less acceleration in the Z direction to avoid unstable walking.

**Figure 11:**
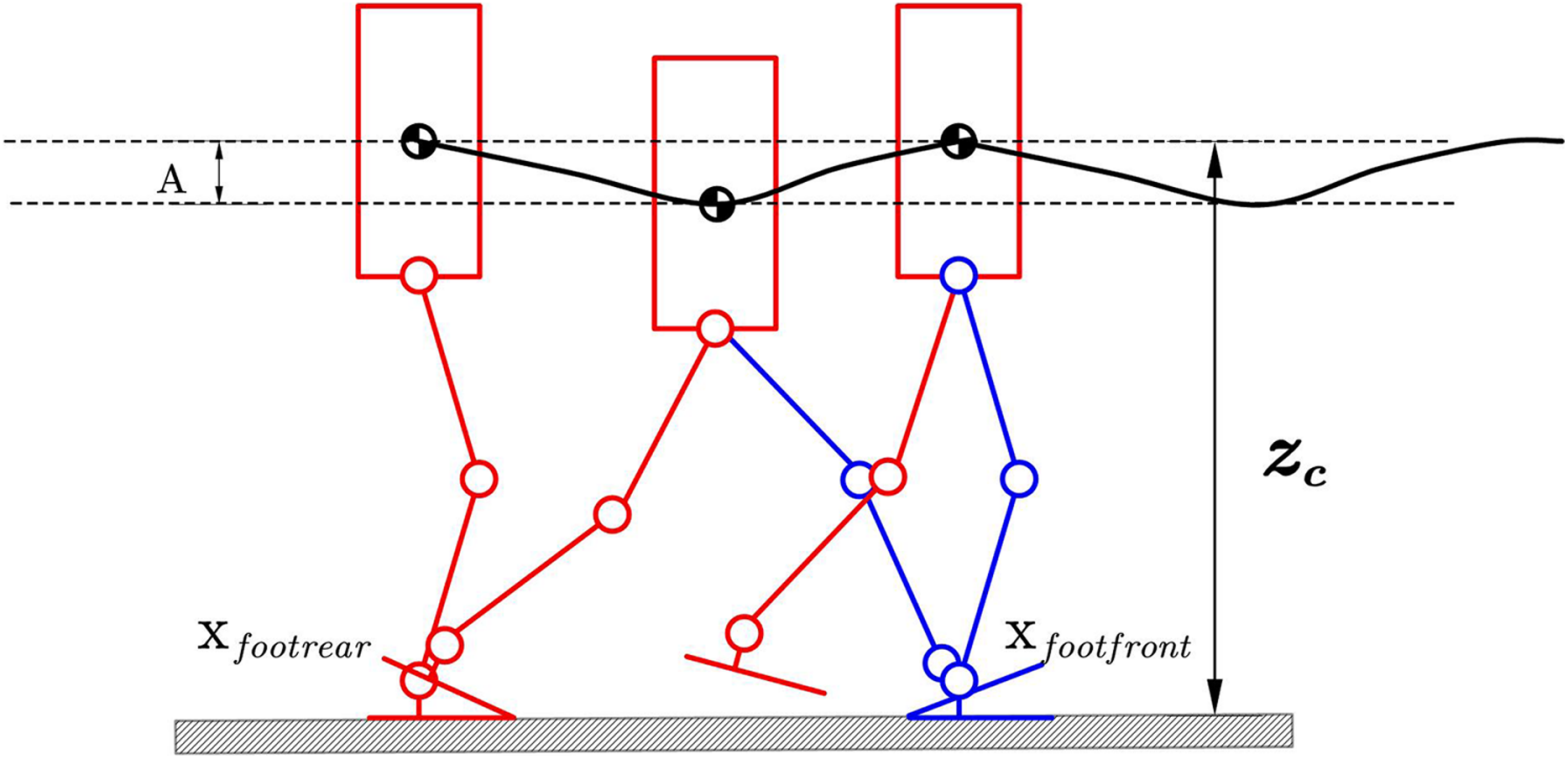
Hip pattern generator.

As show in Fig. 11 we will change the corresponding CoM in the Z direction to satisfy the following expression:

(12) $${Z_{com}} = {Z_c} + A\left[ { - 0.5\tanh {\rm{ }}\left( {4{{{x_{com}} - {x_{footrear}}} \over {{x_{footfront}} - {x_{footrear}}}} - 2} \right) - 0.5} \right]$$

where *x*_*footrear*_ is the position of rear foot. *x*_*footfront*_ is the position of front foot. Respectively, *z*_*c*_ is a constant hip height, and *A* is the amplitude of the specific pattern. A larger *z*_*c*_ means the robot’s knees will extend straighter while walking. The larger *A* means the hip position will be lower while walking, so the knee singularities can be avoided. The parameters *z*_*c*_ and *A* can be changed to generate different walking patterns. We should not let hip-height be a function of time as an independent variable because this will cause the hip height to be discontinuous with respect to time, as the horizontal velocity $\dot x$ is not constant. But our method defines *Z*_*com*_ respect to *x*_*com*_ as in (12). The acceleration caused by the hip motion is:

(13) $$a(t) = - \displaystyle{{2A{L_{step}}} \over {\mathop {\cosh }\nolimits^2 (2 + 4b{L_{step}} - 4{L_{step}}{x_{com}})}}{\dot x_{com}}$$

Compared with the method in the reference (Li et al., 2010), on the one hand, we use a variable height center of mass trajectory instead of the hip joint trajectory so that while completing the human-like gait, it is more in line with the original gait assumptions. At the same time, the center of mass is used. The planning method can also facilitate the design of subsequent controllers. Another convenience is that we use the tanh function instead of the trigonometric function, which is smoother than the trigonometric one.

The centroid trajectory of the robot generated by this generator has the following characteristics:

1. This smooth trajectory reaches the lowest point during the bipedal support phase. This feature allows the robot to have a longer walking step length in the actual walking process. It can also complete the energy conversion through the change of the center of mass to reduce the walking process—power consumption.
2. For continuous acceleration trajectories, the acceleration of the trajectory in the Z direction can be controlled by a reasonable selection of relevant parameters in the trajectory to ensure walking stability.

#### Foot motion generator

In order for the robot to achieve the human-like effect during the walking process, in addition to planning the position trajectory of the foot end, we also need to plan the pitch attitude angle of the foot end during the walking process. The attitude angle adopts five-degree splines in the planning process. The trajectory is planned. The quintic spline trajectory can satisfy the position, velocity, and acceleration information of the starting point and the endpoint at the same time. Moreover, we can plan the trajectory by appropriately selecting the quintic spline curve. First, we distinguish the walking process. It is the bipedal support phase and the single-foot support phase. Separate the posture and position of the foot for planning. Then in the toe-off process, the heel is raised first, in which the posture of the foot determines the ankle trajectory. In the middle process, the posture trajectory of the sole center is the planning target, and the posture of the swinging foot is adjusted to prepare for landing. Then run to the planned landing position in advance and run the corresponding heel-strike after detecting the foot landing. We can define a quintic spline curve by (7) parameters $f\left( {{x_{init}},{x_{end}},{{\dot x}_{init}},{{\dot x}_{end}},{{\ddot x}_{init}},{{\ddot x}_{end}},T} \right)$ which contains the boundary conditions and spline time. The following table gives the boundary conditions of each curve in the entire trajectory planning process as follows:

$$\matrix{ {\matrix{ {toe - off{\kern 1pt} {\kern 1pt} Phase:t \in \left( {0,{T_{toeoff}}} \right)} \hfill \cr {{T_{toeoff}}{\kern 1pt} {\kern 1pt} = {\kern 1pt} {\kern 1pt} {\alpha _{pitch}}{T_{SSP}} + {\beta _{pitch}}{T_{DSP}}} \hfill \cr {{\theta _{foot}} = f\left( {0,{\theta _{toe}},0,0,0,0,{T_{toeoff}}} \right)} \hfill \cr {{x_{foot}}{\kern 1pt} {\kern 1pt} = {\kern 1pt} {\kern 1pt} {L_{toe}}\left( {1 - \cos \left( {{\theta _{foot}}} \right)} \right)} \hfill \cr {{z_{foot}} = {\kern 1pt} {\kern 1pt} {L_{toe}}\sin \left( {{\theta _{foot}}} \right)} \hfill \cr {swing{\kern 1pt} {\kern 1pt} Phase:t \in \left( {{T_{toeoff}},{T_{toeoff}} + {T_{heelstrike}}} \right){\kern 1pt} {\kern 1pt} \left( {{T_{heelstrike}}{\kern 1pt} {\kern 1pt} can{\kern 1pt} {\kern 1pt} be{\kern 1pt} {\kern 1pt} choosed{\kern 1pt} {\kern 1pt} by{\kern 1pt} {\kern 1pt} fsr{\kern 1pt} {\kern 1pt} sensor} \right)} \hfill \cr {{\theta _{foot}} = f\left( {{\theta _{toe}},{\theta _{hell}},0,0,0,0,{T_{SSP}}} \right)} \hfill \cr } }}$$

$${x_{foot}} = f\Bigg( {L_{toe}}\left( {1 - \cos \left( {{\theta _{toe}}} \right)} \right), {L_{stride}} - {L_{heel}}\left( {1 - \cos \left( {{\theta _{heel}}} \right)} \right), $$

$$\displaystyle{{{L_{toe}}\sin \left( {{\theta _{toe}}} \right){\theta _{toe}}} \over {{\alpha _{pitch}}{T_{SSP}}}},\displaystyle{{{L_{heel}}\sin \left( {{\theta _{heel}}} \right){\theta _{heel}}} \over {\left( {1 - {\alpha _{pitch}}} \right){T_{SSP}}}}z,0,0 \Bigg) $$

(14) $$\matrix{ {\matrix{ {heel - strike{\kern 1pt} {\kern 1pt} Phase:} \hfill \cr {{\theta _{foot}} = f\left( {{\theta _{hell}},0,0,0,0,0,2{\alpha _{DSP}}{T_{DSP}}} \right)} \hfill \cr {{x_{foot}} = {\kern 1pt} {\kern 1pt} {L_{heel}}\left( {1 - \cos \left( {{\theta _{heel}}} \right)} \right)} \hfill \cr {{z_{foot}} = {L_{heel}}\sin \left( {{\theta _{heel}}} \right)} \hfill \cr } } \hfill \cr }$$

where *α*_*pitch*_ *α*_*DSP*_ and *β* are trajectory parameters which can define the detail motion of foot.

**Figure 12:**
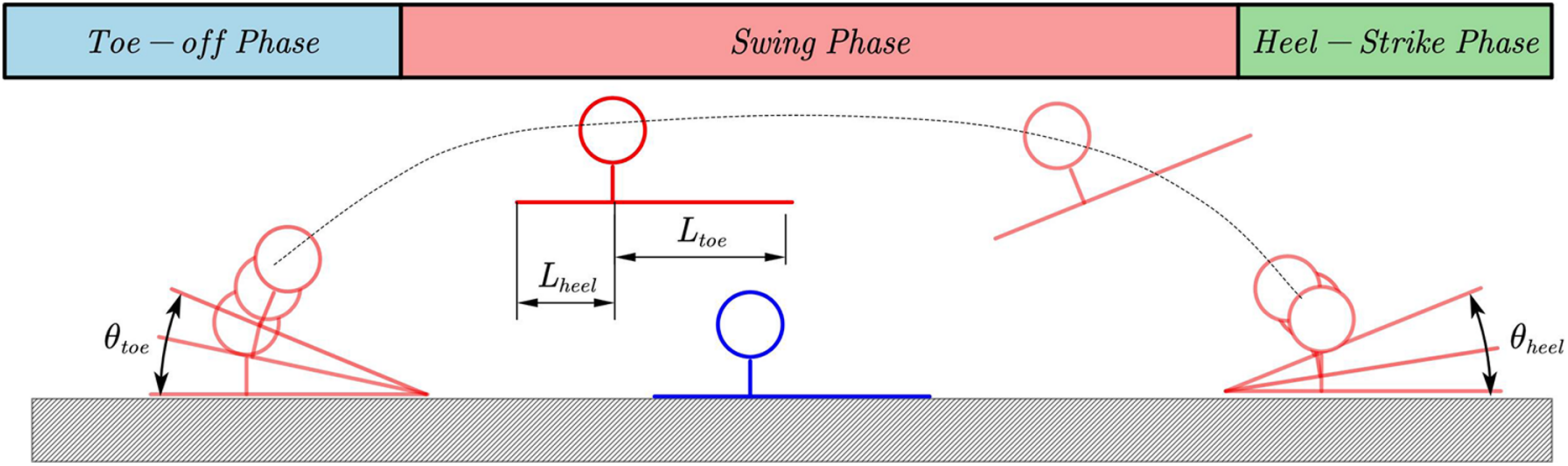
Pattern of human motion and posture in a typical walking cycle.

Figure 12 show the whole curve of foot and some specific parameters in (14).

## Stablizer

### Event based walking phase switcher

When the robot performs bipedal walking, the two legs alternately execute the trajectory of the landing phase and air phases trajectory. Ideally, after the supporting leg finishes the ground phase trajectory, it should immediately switch to the air phase trajectory. The swing leg is the opposite. After executing the vacant phase trajectory, switch to the ground phase trajectory immediately. Furthermore, the switching between the two should be completed at the same instant. However, in the actual execution process, if the open-loop control is adopted for the phase switching time, the actual switching timing of the two legs always has a deviation before and after. The greater the deviation before and after the switching timing, the greater the deviation caused by the center of mass trajectory, and the more it affects gait execution. In order to improve this phenomenon, this article adopts the method of installing pressure sensors on the soles of the feet to control the phase switching time of the two legs to reduce the vertical movement of the torso and the impact of the swing leg when performing the planar biped gait. Ensure the smooth running of the bipedal gait.

**Figure 13:**
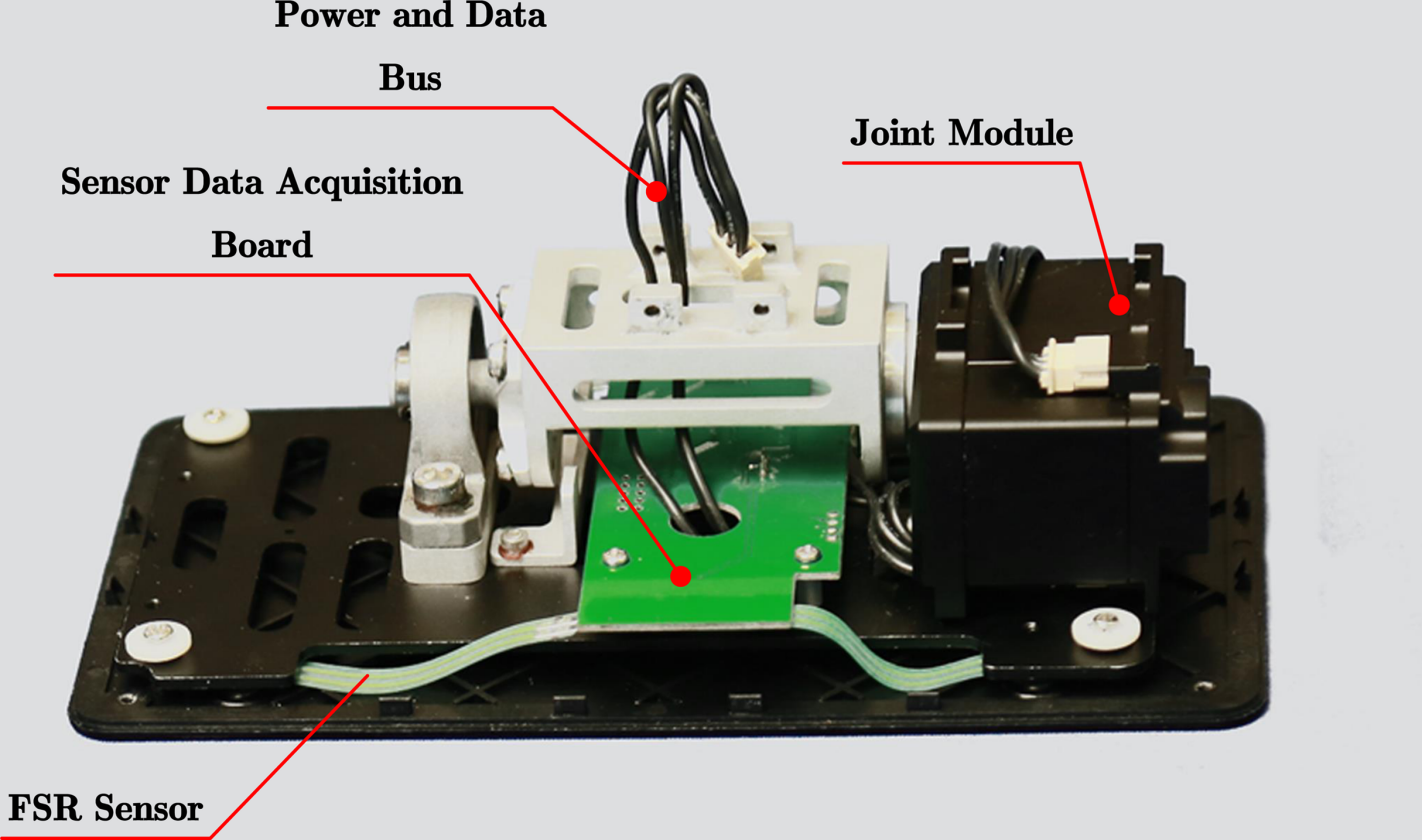
FSR installed on the robot.

**Figure 14:**
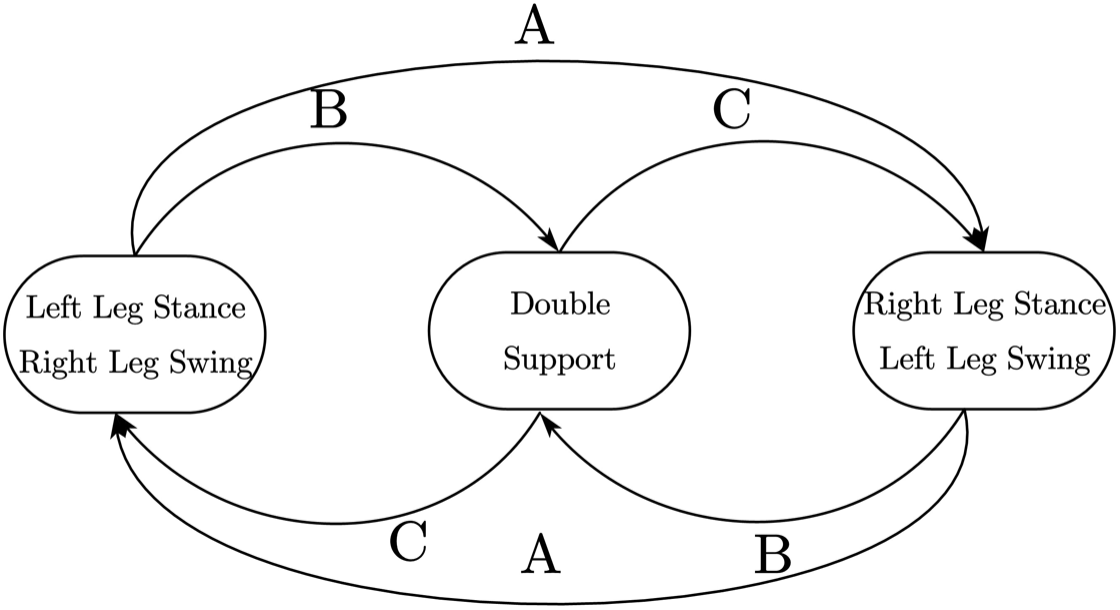
Walking phase state machine.

As shown in Fig. 13, a resistive film pressure sensor is installed on each of the four corners of each foot. When the sole of the foot touches the ground, the sensor gives a contact force signal. When the sensor detects that the contact force is greater than 1N, it is considered that the pressure sensor has detected the ground signal. When the robot is walking, the sole of the foot may not be completely flush with the horizontal surface when touching the ground. Therefore, when the same sole of the foot is on the ground, the four sensors on it may not detect the ground signal. Experiments have found that when performing bipedal plane gait, the switching time is advanced, usually near the time point of switching from the air phase trajectory to the ground phase trajectory. At least three pressure points are detected for more than two consecutive seconds sampling periods. When the ground is detected, it is considered that the leg has touched the ground. Switch directly to the ground phase trajectory. For the case of switching time lag, it is generally near the time point when the trajectory of the ground phase is switched to the trajectory of the air phase. At this time, the grounding situation of the other leg should be used as the basis for switching judgment. In order to ensure the regular switching of the landing phase of the legs, the finite state machine needs to be used to control the landing status of the legs so that it only jumps under several normal landing conditions, thereby avoiding falling to the ground. The landing condition finite state machine is shown in Fig. 14.

Among them, A represents the state switching route when the gait is running normally, B represents the state switching route when the vacant leg advances or lags behind the ground. C represents the state switching route adjusted by the program when the feet are on the ground. The exact landing time and landing state of the robot can be obtained through pressure sensitivity. This information can be used to adjust the gait parameters in real-time to execute the planned trajectory, thereby avoiding the problem of unsynchronized phase switching of the two feet landing on the ground during open-loop control and can enhance walking stability.

#### Upper body feedback controller

In the previous section, we have analyzed the impact of the introduction of the human-like gait planning framework on the robot’s stability when it is walking. During the robot’s operation, we will find that the error in the operation of the robot’s joint unit and the error in the mechanical structure of the robot itself will cause The actual ZMP point to have a specific offset. In order to solve this problem, the solution of a scenario is to use the ZMP control of the ankle joint, but the modular actuator used in the child-size robot cannot directly control the force. Therefore, This paper proposes a method to achieve force control under the constraints of using modular actuators. The principle of ankle joint force control to compensate for the deviation of the ZMP point is relatively simple, which can be deduced according to the relevant theory of LIPM:

(15) $$\ddot x = \displaystyle{g \over {{z_c}}}x + \displaystyle{1 \over {M{z_c}}}\tau$$

Moreover, we use the angular velocity of the torso to approximate the torso velocity, and the torso velocity will cause changes in the angular momentum of the torso, which will affect the ZMP point of the plantar. Therefore, we need to adjust the torque applied on the ankle joint to avoid changing the plantar ZMP point. The problem causes instability. However, we use a modular-based robot to drive the joints, so we cannot directly give torque commands to the ankle joint. Nevertheless, through the analysis of the following modular joint control framework, we can see that we can use some methods to give position commands so that the ZMP point can be kept within a stable range. For the control frame of the modular drive joint, it can be found that the current information is given by the difference between the current position and the given position. Moreover, before the controller is not attached, The given joint angle trajectory can be regarded as continuous, and the joint tracking characteristics of the robot itself are good. We regard the reference position and the actual position as approximately the same. We need to add a different value to the original reference angle information for the ankle joint torque command because this difference value will become an incremental current data after passing through the position controller. To achieve the effect we need on the lower price joint unit controller. The specific controller form is as follows:

(16) $${\theta _{goal}} = {\theta _{ref}} + {K_p}*{\dot \gamma _{body}}$$

where *γ*_*body*_ is the angular speed of pitch of body. The parameter *K*_*p*_ need to be adjusted carefully to avoid toe shaking during walking.

## Experiments

### Simulations

**Figure 15:**
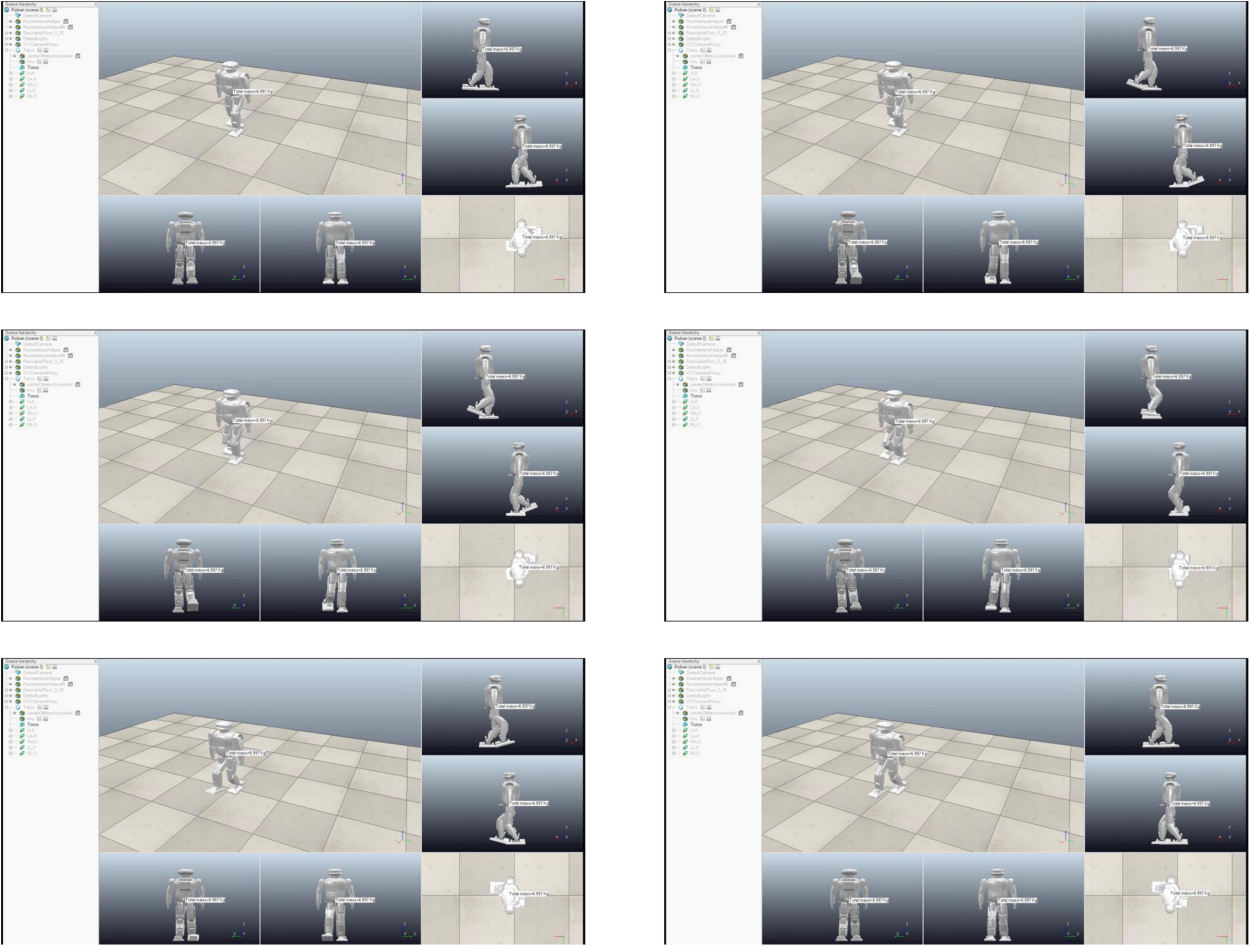
Humanoid walk scene in V-rep.

Figure 15 shows the scene of the robot walking in the simulation environment. It can be found that the robot can achieve Toe-off, heel-contact walking in the simulation environment.

**Figure 16:**
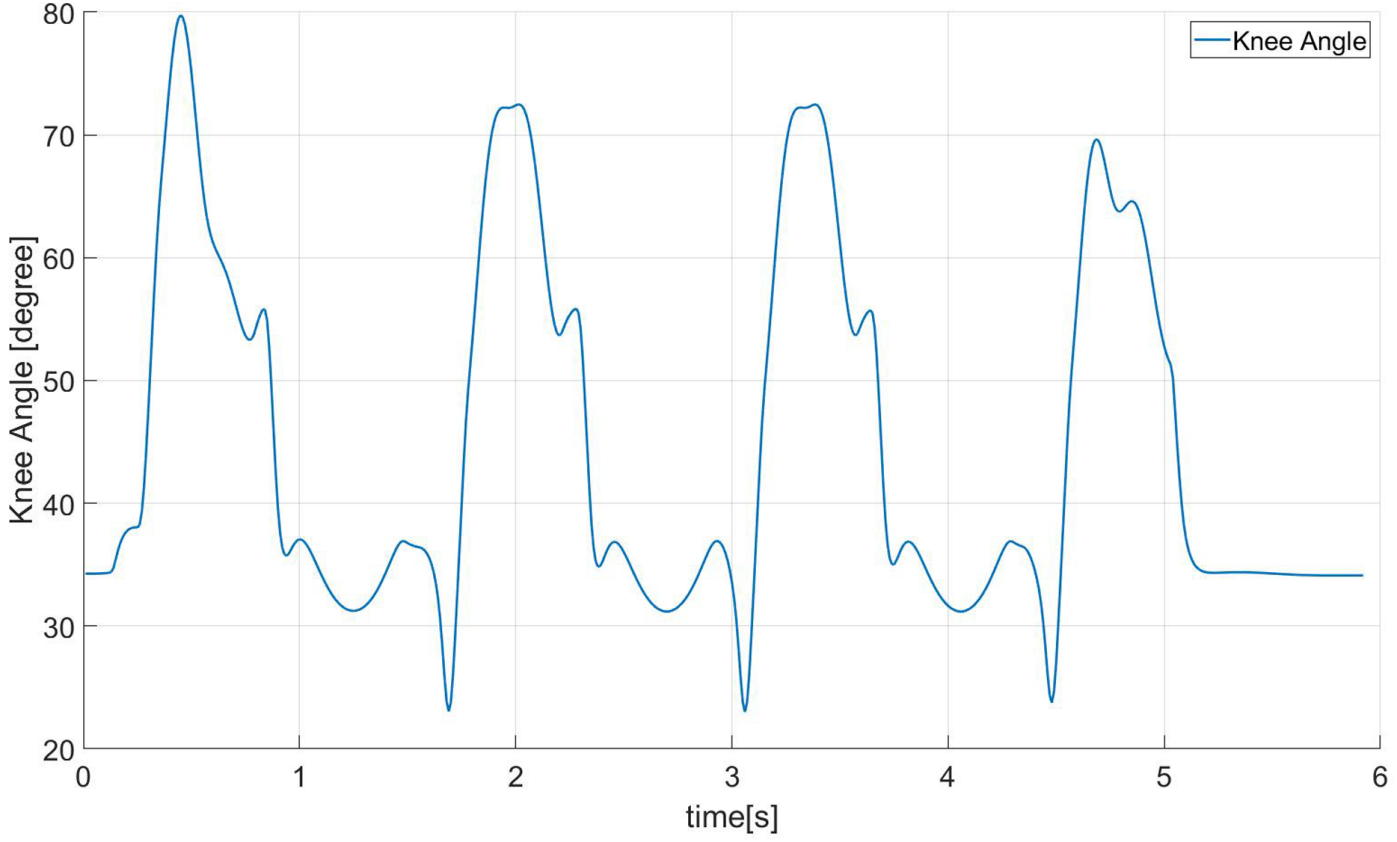
Robot knee angle.

Figure 16 shows the trajectory of the knee joint angle when the robot is walking in the simulation environment. Generally speaking, the change of the knee angle when a human is walking is between 0–60 degrees. The minimum angle of the knee joint of the robot running under the human-like walking frame we realized is about 20 degrees, and in order to achieve a longer step length, the maximum angle is about 70 degrees.

**Figure 17:**
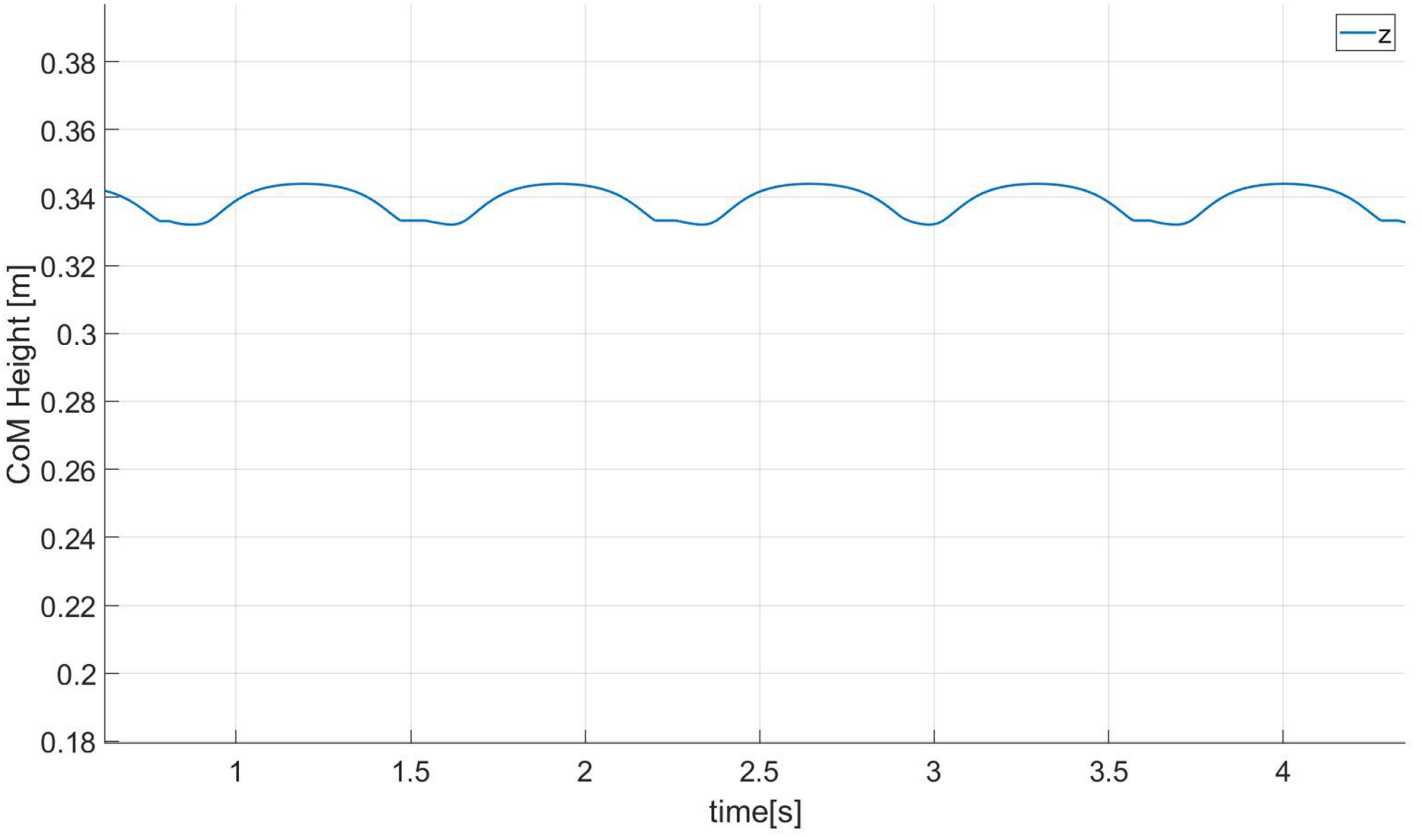
CoM height.

Figure 17 shows the height curve of the center of mass in the z direction when the robot is walking in the simulation environment. It can be found that the height change of the center of mass in the z direction is about 1.7 cm, and this change in the height of the center of mass can make the robot’s new walk more huan-like effect.

We can evaluate the anthropomorphism of biped robot walking from the following aspects: The first feature of human walking is to achieve a larger step length and thus have a “single toe support stage”. From Fig. 15, we can It can be seen that our robot has the above characteristics. Secondly, humans have a tendency to straighten their knees during walking. It can be seen from Fig. 16 that the minimum bending angle of the robot knee in the simulation environment can reach 20 degrees, while the minimum knee of the robot using a standard gait algorithm The angle will basically be above 40 degrees. The third aspect is that the height of the torso (center of mass) will change when humans walk, thereby increasing the step length of a single step. From Fig. 17, we can find that our method has this feature.

#### Real robot experiments

**Figure 18:**
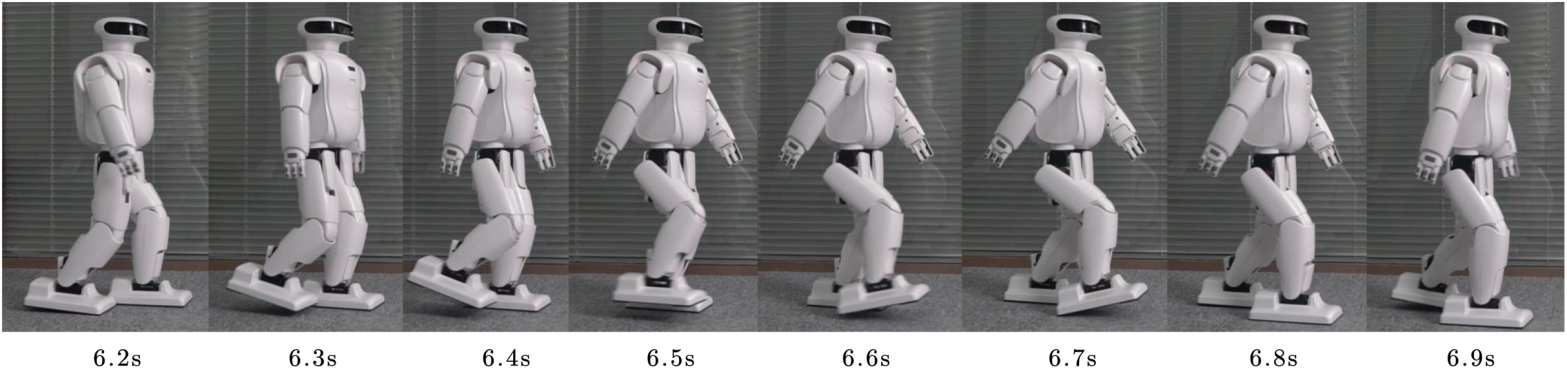
Humanoid walking in real scene.

We have implemented the suggested walk controller on the Roban child-size humanoid robot, which is 68 cm height, weights 6.5 kg. It has Core i3 processor for the onboard processing, has various sensors including IMU, FSR on foot nd joint encoders at the joints It can be seen from Fig. 18 that a real robot can achieve a better human-like walking effect on flat ground.

**Figure 19:**
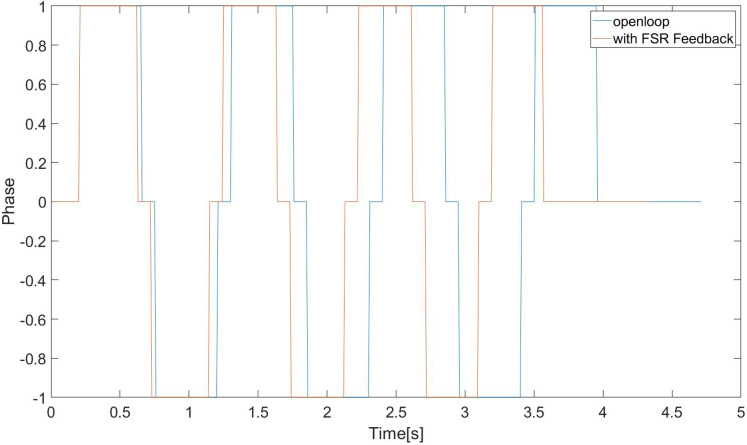
Phase switch in real scene.

We compared whether to use an event-based trajectory switching controller. From Fig. 19, it can be found that if the controller we proposed before is not used, the running time of each step of the robot is the same, but in the actual running process, the swing phase will land in advance, which will affect the stability of the robot. However, the controller we proposed can switch the working state of the state machine according to the actual landing situation, thereby improving the walking effect of the robot.

**Figure 20:**
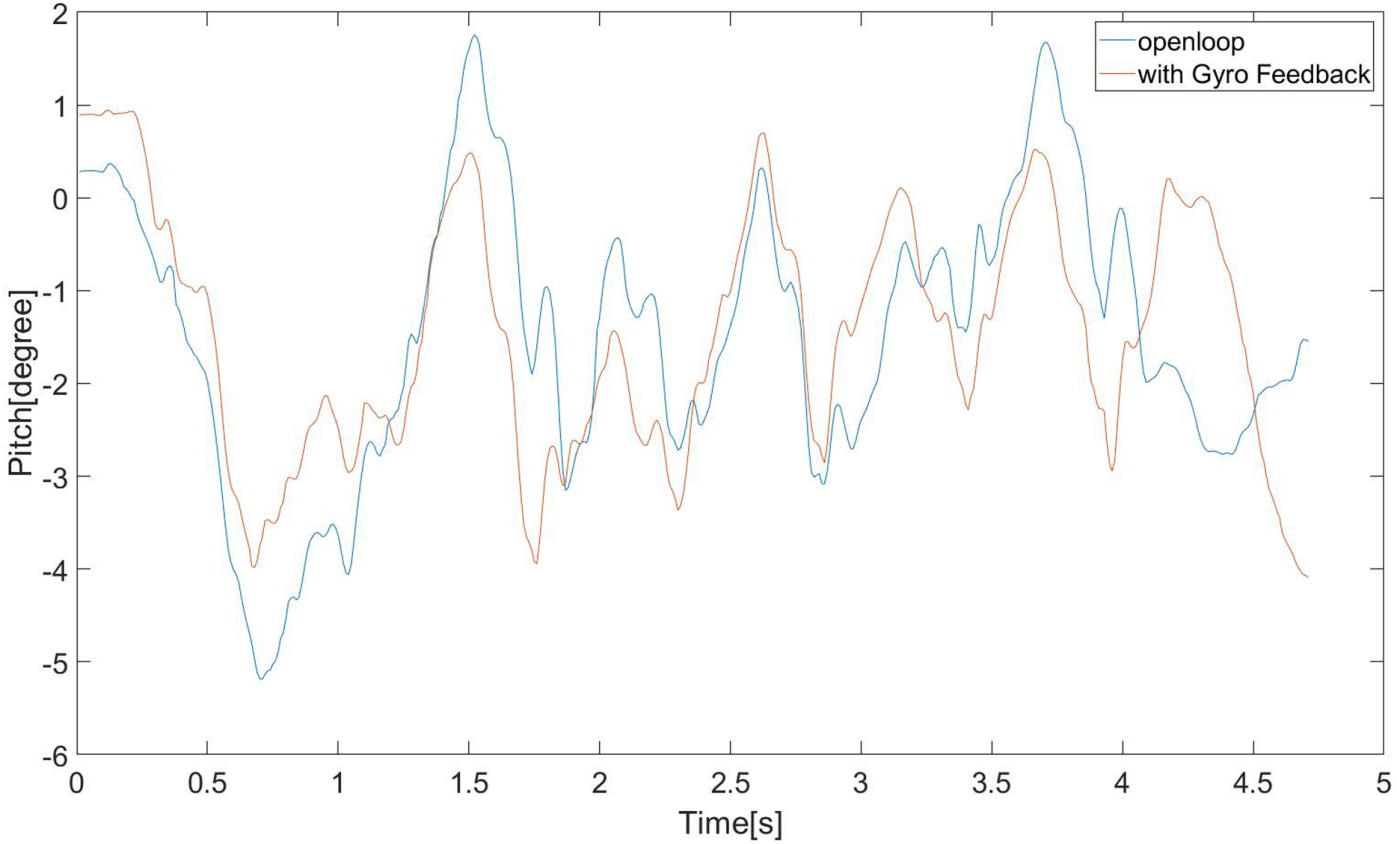
Robot’s pitch vibration in real scene.

We compared whether to use Upper Body Feedback Controller. From Fig. 20, it can be found that the swing amplitude of the robot’s upper body is relatively large and inconsistent when the controller is not used. However, when the controller we proposed is used, the swing of the robot torso in the pitch direction is small, and The amplitude is consistent, which is conducive to the stability of walking.

## Conclusions

In this paper, a human-like walking control framework based on DCM com planning is proposed. By using capture point theory, we can get the com trajectory in the horizon plane. By constructing a reasonable center of mass Z direction and foot end trajectory generator, makes the walking step length of the robot lengthen (in this experiment, the strike reaches 75% of the leg length). It can also make the knee joint of the robot closer to the straight state so that the robot can be anthropomorphized during walking. At the same time, to avoid the influence of our human-like gait planning method on the walking stability of the robot itself, we designed an event-based trajectory switching controller and a trunk stability controller to ensure the robot’s stability during walking. We also used our method to conduct experiments on the dynamic simulation environment and child-size actual robots. The simulation and physical robots both achieve relatively stable walking on flat ground. The current control framework allows the robot to walk on flat ground. In our subsequent research, we can consider modifying the current control framework so that the robot can also walk on a human-like gait on uneven roads. Additionally, the introduction of a human-like gait does not resolve the problem of stability for the robot. The instability of the overall walking process is aggravated, and the stability controller for the human-like gait could be further designed to improve the stability of the robot’s walking gait.

## Supplemental Information

10.7717/peerj-cs.797/supp-1 Supplemental Information 1 Walk simulation raw data.

10.7717/peerj-cs.797/supp-2 Supplemental Information 2 Source Code.

## Additional Information and Declarations

### Data Availability

The following information was supplied regarding data availability: Raw data are available in the Supplemental File.
